# Bilosomes as a promising nanoplatform for oral delivery of an alkaloid nutraceutical: improved pharmacokinetic profile and snowballed hypoglycemic effect in diabetic rats

**DOI:** 10.1080/10717544.2022.2110997

**Published:** 2022-08-16

**Authors:** Mohammed H. Elkomy, Hussein M. Eid, Mohammed Elmowafy, Khaled Shalaby, Ameeduzzafar Zafar, Mohamed A. Abdelgawad, Mostafa E. Rateb, Mohammed R.A. Ali, Izzeddin Alsalahat, Heba A. Abou-Taleb

**Affiliations:** a Department of Pharmaceutics, College of Pharmacy, Jouf University, Sakaka, Saudi Arabia; b Department of Pharmaceutics and Industrial Pharmacy, Faculty of Pharmacy, Beni-Suef University, Beni-Suef, Egypt; c Department of Pharmaceutical Chemistry, College of Pharmacy, Jouf University, Sakaka, Saudi Arabia; d School of Computing, Engineering & Physical Sciences, University of the West of Scotland, Paisley, UK; e Department of Pharmacology and Toxicology, Faculty of Pharmacy, Beni-Suef University, Beni-Suef, Egypt; f UK Dementia Research Institute Cardiff, School of Medicine, Cardiff University, Cardiff, UK; g Department of Pharmaceutics and Industrial Pharmacy, Faculty of Pharmacy, Merit University (MUE), Sohag, Egypt

**Keywords:** Berberine, diabetes mellitus, bilosomes, bioavailability, pharmacokinetics, bile salts, optimization

## Abstract

Diabetes mellitus is a life-threatening metabolic disease. At the moment, there is no effective treatment available to combat it. In this study, we aimed to develop berberine-loaded bilosomes (BER-BLS) to boost the oral bioavailability and therapeutic efficacy of berberine, a natural antidiabetic medication. The BER-BLS was fabricated using a thin-film hydration strategy and optimized using a central composite design (face-centered). The average vesicle size, entrapment efficiency, and surface charge of the optimized BER-BLS preparation were 196.5 nm, 89.7%, (−) 36.4 mV, respectively. In addition, it exhibited higher stability and better-sustained release of berberine than the berberine solution (BER-SOL). BER-BLS and BER-SOL were administered to streptozocin-induced diabetic rats. The optimized BER-BLS formulation had a significant hypoglycemic impact, with a maximum blood glucose decrease of 41%, whereas BER-SOL only reduced blood glucose by 19%. Furthermore, the pharmacological effect of oral BER-BLS and BER-SOL corresponded to 99.3% and 31.7%, respectively, when compared to subcutaneous insulin (1 IU). A pharmacokinetic analysis found a 6.4-fold rise in the relative bioavailability of berberine in BER-BLS when compared to BER-SOL at a dosage of 100 mg/kg body weight. Histopathological investigation revealed that BER-BLS is suitable for oral administration. Our data demonstrate that BLS is a potential nanocarrier for berberine administration, enhancing its oral bioavailability and antidiabetic activity.

## Introduction

Diabetes mellitus (DM) prevalence increases due to poor nutrition and lifestyle behaviors, imposing a significant burden on individual families and societies (Karthikeyan et al., 2014). Currently, DM is generally treated with insulin injections and oral antidiabetic medications. However, subcutaneous insulin injections are painful and are sometimes linked with an allergic response, lipodystrophy, hypoglycemia, and even hyperinsulinemia (Wong et al., 2016). Similarly, oral antidiabetic medications such as glipizide, metformin, and repaglinide have gastrointestinal side effects, a significant risk of hypoglycemia, and weight gain (Arai et al., 2016). While these therapeutic approaches may improve some of the symptoms associated with DM, they cannot cure the disease completely. New effective drugs for DM treatment are still essential.

Oral administration of drugs is the most effective way for patients, particularly those with chronic conditions. Despite the many advantages of oral administration, some medications suffer from low oral bioavailability. This might be related to first-pass metabolism, limited drug solubility and permeability, and drug efflux (Arzani et al., 2015). Dissolution is the rate-limiting step for poorly soluble drugs, resulting in inconsistency in absorption and limited bioavailability. Many attempts have been utilized to boost the oral bioavailability of drugs (Kawabata et al., 2011; Zhang et al., 2013).

Recently, there has been a rise in interest in investigating nanocarriers for oral administration since they offer several benefits over traditional dosage forms (Zhang et al., 2013). Additionally, these nanocarriers are known to improve dissolution rates of poorly soluble drugs and effectively circumvent first-pass metabolism through lymphatic transport stimulation, resulting in increased bioavailability (Nishioka & Yoshino, 2001; Ahad et al., 2018). The biological fate of vesicular systems has been demonstrated to be influenced by adding charge-inducing agents and bile salts after oral delivery (Roger et al., 2010).

Bilosomes (BLS) are lipid nanovesicles enriched by bile salts with a superior ability to penetrate biological membranes (Elkomy et al., 2022). Bile salts hinder the breakdown of nanocarriers in the gastrointestinal tract (GIT), hence improving penetration, which is advantageous for oral administration (Waglewska et al., 2020; Elkomy et al., 2022). Furthermore, the inclusion of certain bile salts, such as sodium deoxycholate (SDC), improves the colloidal stability (Elkomy et al., 2022). Additionally, BLS has a nanoscale diameter and a fluidizing action, which contributes to its absorption enhancement (Al-Mahallawi et al., 2015; Ahmed et al., 2020). Interestingly, bile salts are commonly used as a penetration enhancer, resulting in high permeability across biological barriers (Arzani et al., 2015). Besides, the existence of bile salts in lipid bilayers may help stabilize the nanovesicles, enabling them to tolerate the distracting effects of physiological acids in the GIT (Aburahma, 2016). It has been found that BLS enhance vesicle uptake by intestinal epithelial cells and inhibit enzymatic activity at the absorption location. Numerous efforts have been made to augment the oral bioavailability of different medications by using BLS (Hu et al., 2013; Niu et al., 2014; Li et al., 2017; Fan et al., 2018), notably those containing SDC (Guan et al., 2011; Ahad et al., 2018; Elnaggar et al., 2019).

Berberine (BER), an isoquinoline alkaloid obtained from Coptis Chinensis, is commonly used in Asian countries to treat GIT infections and other inflammations. It has recently been shown to have therapeutic implications for type 2 diabetes therapy (Lee et al., 2006; Yin et al., 2008; Tian et al., 2016; Chen & Yang, 2017). BER may influence islet function in DM by reducing oxidative stress through the miR-106b/SIRT1 pathway (Panda et al., 2021). In diabetes patients, BER has been reported to promote insulin sensitivity and boost lipid and glucose metabolism by activating adenosine monophosphate-activated protein kinase (Yin et al., 2012). Additionally, BER may enhance glucosidase inhibition and control numerous signaling pathways by targeting noncoding RNA, exerting an antidiabetic impact (Jhong et al., 2015; Chang, 2017). However, therapeutic usage of BER is restricted owing to its poor intestinal absorption after oral dosing (Abo El-Enin et al., 2022). Additionally, oral treatment, particularly at high doses, often results in GIT discomfort due to significant exposure to free BER (Wu et al., 2014). As a result, it is required to create enhanced formulations of BER to increase its oral delivery effectiveness and hence its antidiabetic impact in vivo.

This study evaluates the effectiveness of BLS in improving BER oral bioavailability. To our knowledge, this is the first study to explore the potential of bilosomes as a nanocarrier for BER delivery through the oral route. The formulations were optimized using a central composite design (face-centered) and then characterized for *in vitro* release, morphology, stability, and histopathological examination. Finally, the optimal BER-BLS formulation was assessed for pharmacokinetic behavior and *in vivo* antidiabetic activity in a streptozotocin-induced diabetic rat model.

## Materials and methods

### Materials

Berberine chloride, Soybean phosphatidylcholine (SPC), Cholesterol, Sodium deoxycholate (SDC), dialysis bags (MW cut off: 12 kDa), Streptozocin, Chloroform (HPLC), Methanol (HPLC), and Acetonitrile (HPLC) were procured from Sigma-Aldrich (St. Louis, MO, USA). The other chemical substances and solvents used were of analytical quality.

### Experimental design and optimization

BER-BLS formulations were optimized using a central composite design (face-centered) with three central points. The independent variables were SPC (molar concentration), SDC amount (mg), and cholesterol amount (mg). The values of each independent variable were determined based on preliminary experiments and described as three levels. Seventeen formulations were conducted, fourteen of which included the experimental runs and three center points. The dependent variables that were examined were vesicle size (VS), zeta potential (ZP), and entrapment efficiency (EE). Experimental data were analyzed using Design-Expert® software version 12.0.3.0 (Stat-Ease, Inc., Minneapolis, MN, USA) to independently source the major impacts of these components, followed by ANOVA to assess the significance of each factor. The plot3D package in the R program was used to create 3D response surface plots (Soetaert, 2017). The formulation factors and components of the BER-BLS developed using the central composite design are illustrated in Tables 1 and 2. Then, the desirability of selecting an optimum formula was determined.

**Table 1.** Design of independent and dependent variables utilized for bilosomal formulation optimization, and their levels.

	Levels		
Independent variables	−1	0	1
*X*_1_: SPC (molar concentration)	0.03	0.045	0.06
*X*_2_: SDC amount (mg)	15	22.5	30
*X*_3_: Cholesterol amount (mg)	10	15	20
Dependent variables			
Vesicle size	Minimize		
Zeta potential	Maximize (as absolute value)		
Entrapment efficiency	Maximize		

**Table 2.** Experimental runs as stated by the central composite design and associated values of all responses.

F	*X*_1_: SPC (molar concentration)	*X*_2_: SDC amount (mg)	*X*_3_: Cholesterol amount (mg)	*Y*_1_: VS (nm)	*Y*_3_: ZP (mV)	*Y*_2_: EE (%)
1	0.06	22.5	15	164.2 ± 3.4	(−)33.8 ± 1.9	88.2 ± 3.7
2	0.03	15	20	225.7 ± 4.7	(−)24.5 ± 2.3	90.2 ± 4.1
3	0.06	30	20	239.3 ± 3.6	(−)39.5 ± 3.6	92.4 ± 4.5
4	0.03	30	10	141.3 ± 2.3	(−)37.1 ± 1.8	69.4 ± 3.2
5	0.03	22.5	15	179.2 ± 2.9	(−)30.1 ± 1.5	78.3 ± 3.6
6	0.045	15	15	184.2 ± 3.5	(−)25.7 ± 1.2	75.5 ± 2.9
7	0.045	22.5	20	211.4 ± 3.2	(−)33.5 ± 2.5	94.8 ± 4.7
8	0.03	15	10	127.4 ± 2.5	(−)24.3 ± 2.8	65.3 ± 2.5
9	0.06	30	10	134.6 ± 3.7	(−)37.8 ± 3.1	71.5 ± 3.2
10	0.045	22.5	10	109.1 ± 2.1	(−)31.4 ± 2.4	72.9 ± 3.7
11	0.03	30	20	263.2 ± 3.6	(−)36.7 ± 2.6	90.3 ± 4.6
12	0.045	22.5	15	171.6 ± 3.8	(−)32.1 ± 3.5	85.3 ± 3.2
13	0.045	30	15	195.1 ± 2.9	(−)36.4 ± 3.3	76.3 ± 2.9
14	0.045	22.5	15	175.3 ± 1.7	(−)31.7 ± 2.4	86.7 ± 3.8
15	0.045	22.5	15	173.9 ± 3.2	(−)31.6 ± 3.1	84.9 ± 3.9
16	0.06	15	10	115.9 ± 2.3	(−)26.2 ± 1.7	67.3 ± 2.4
17	0.06	15	20	218.5 ± 3.6	(−)25.9 ± 1.9	91.4 ± 5.2

### Formulation of berberine-loaded bilosomes

BER-BLS was prepared using a thin-film hydration procedure with minor modifications (Elkomy et al., 2022). Briefly, SPC, BER (10 mg), and cholesterol were dissolved in a chloroform–methanol combination (10 mL, 2:1, v/v) in a round-bottom flask. The organic phase was evaporated via a rotating evaporator (Stuart rotary evaporator, United Kingdom) at 40 °C under reduced pressure, resulting in a thin dry layer (Eid et al., 2022). Following evaporation of the organic phase residue, the film was rehydrated with 10 mL phosphate buffer saline (pH 7.4) containing SDC. A bath sonicator (Ney ultrasonic cleaner, USA) was used to sonicate the resultant hydrated dispersion of bilosomes (2 rounds of 5 minutes with a 5 min interval between each cycle). Finally, the formulations were refrigerated (4 °C) for further evaluation.

### Chromatographic conditions

The quantity of BER was determined using a validated HPLC method (Wang et al., 2014). Agilent Eclipse C18 column (i.d., 5 μm PS, 4.60 mm × 25 cm) was used to estimate BER concentration. A mobile phase consisting of acetonitrile and 0.05 mol/L NaH_2_PO_4_ (30:70, pH adjusted to 2.5 with phosphoric acid) was pumped through the HPLC apparatus at a 1 mL/min flow rate. UV detection was performed at 345 nm and 30 °C. The injection volume was 20 µL, and the retention duration was 5 min. The HPLC method was very sensitive and produced a linear calibration graph with a concentration range of 0.01–1 µg/mL (*R*^2^ = 0.999).

### BER-BLS characterization and optimization

#### Vesicle size and zeta potential evaluation

Vesicle size and zeta potential were analyzed using a dynamic scattering technique (Zetasizer Nano ZS, UK). The different formulations were mixed with distilled water (1:10) (Eissa et al., 2022) before measurement and were analyzed at 25 °C (Eid et al., 2021).

#### Berberine entrapment efficiency

Entrapment inside the bilosomal preparation was estimated indirectly by subtracting the non-entrapped amount (free BER) from the amount initially added (10 mg) (Abo El-Enin et al., 2022; Elkomy et al., 2022). The dispersion was centrifuged via a cooling centrifuge (SIGMA 3-30 K, Steinheim, Germany) at 14,000 rpm (3 h, 4 °C) to separate the supernatant containing unentrapped BER (Elkomy et al., 2017). The supernatant was diluted, and the BER quantity was determined using the HPLC method. The following equation was used to calculate EE% (Elkomy et al., 2021):

(1) $$\text{EE \%}=\frac{\text{Total amount of BER }-\text{amount in supernatant}}{\text{Total amount of BER}}\times 100$$

### Optimized BER-BLS characterization

#### Transmission electron microscopy

The morphology and VS of the optimized BER-BLS formulation were examined using a transmission electron microscope (JEOL JEM-1400, Japan) operating at 80 kV (Eid et al., 2019). The optimized BER-BLS formulation was diluted 50-fold with distilled water, applied on a copper grid (300 mesh), and left for 10 min to dry. One drop of phosphotungstic solution (1.5% w/v) was added to the copper grid after drying and allowed to dry at 25 °C (Elkomy et al., 2016).

#### In vitro release study

Vertical Franz cells (5 cm^2^ diffusion area) were used to conduct an *in vitro* release study. In the donor chamber, the optimized l BER-BLS formulation and BER-SOL were placed (equivalent to 3 mg BER). The receptor media consisted of 100 mL freshly prepared simulated intestinal fluid (SIF; pH = 6.8) stirred at 100 rpm at 37 ± 0.5 °C. The dialysis membrane (soaked in receptor medium for 24 h) separated the donor chamber from the receptor one (Eid et al., 2019). One milliliter was collected from the receptor chamber at predefined time intervals and replaced with one mL of fresh SIF. The collected samples were filtered, and the BER quantity was evaluated using the HPLC method in order to calculate the percentage of cumulative drug released using the equation below:

$\text{Cumulative drug released \%}=\frac{Qi}{Qr}\times 100$(2)

*Qi* and *Qr* are the cumulative drug released at time intervals and the initial amount of BER initially applied (3 mg), respectively (Aboud et al., 2018).

#### Stability study

A stability study was performed on the optimized formulation by storing it in a glass vial at 4 °C for 90 days (Eid et al., 2019; Elkomy et al., 2022). After formulating the optimized BER-BLS, samples were taken at predetermined intervals for 90 days. The VS, EE, and ZP values of the samples obtained were determined (Elkomy et al., 2017).

### In vivo experiments

Wistar male rats (220 ± 20 g) were used for in vivo investigations. All animal experiment procedures were approved by the Animal Ethics Committee at Beni-Suef University (Acceptance No: 022-259) and were performed in accordance with the guidelines of the Declaration of Helsinki.

#### Hypoglycemic effect in diabetic rats

Streptozotocin-induced diabetic rats were used to assess the hypoglycemic impact of different formulations. The approach for induction was as described before (Eid et al., 2022; Panda et al., 2021). The diabetic rats were split into five groups (each of which had six rats): saline, empty bilosomes (BLS), BER-SOL, optimized BER-BLS, and insulin solution. The rats were fasted overnight before and throughout the experiment, but they had unlimited access to water. The rats were either intragastrically given saline (negative control), BLS, BER-SOL, and BER-BLS, or subcutaneously given insulin solution (positive control). The formulations containing BER were administered orally at a dose of 100 mg/kg, while insulin was administered subcutaneously at a dose of 1 IU/kg. For the blood glucose level (BGL) test, blood samples were obtained from the tail veins of rats before and after drug administration at specified time intervals. The BGLs were measured using a commercial glucometer (GlucoDr®, Gyeonggi-do, Korea). The pharmacological effect (PE) of oral preparations was determined in comparison to insulin (S.C.) utilizing the area above the curve (AAC) of the BGL curve using the following equation:

$$\mathrm{PE}(\%)=\frac{\mathrm{AAC}(i.g.)}{\mathrm{AAC}(s.c)}\times 100$$

Following each BGL test, rats receiving BER-SOL or BER-BLS were immediately sedated with ether inhalation, and blood samples were collected for drug bioavailability and pharmacokinetics studies. The groups that received normal saline (negative control) and optimized BER-BLS were used in the histological evaluation to determine the safety of the bilosomal system on the intestinal mucosa.

#### Bioavailability and pharmacokinetic study

The two groups of rats mentioned above (BER-SOL and BER-BLS) were used to assess the bioavailability and pharmacokinetics of BER formulations, and blood samples were collected into heparinized tubes from the tail vein at preset time intervals after oral administration. The plasma was then separated from the blood by centrifugation; the proteins were precipitated by adding acetonitrile (1:5) and vortexed for 3 min to ensure complete mixing. The mixture was centrifuged for 25 min at 11,000 rpm, and the supernatant was evaporated to dry at 37 °C. The residues were reconstituted in 150 μL of the mobile phase. Afterward, 20 µl was pumped into the HPLC system. A non-compartmental analysis function coded into R was used to analyze the pharmacokinetic data according to a previously published method (Elkomy, 2020).

#### In vivo histopathological study

Histological examinations were conducted to determine the possibility of morphological alterations and damage to the intestine induced by BLS. After 24 h, the histopathological analysis was performed on the normal saline group and the optimized BER-BLS formulation from the previously described groups. Tissue samples from various sections of the small intestine were added to buffered formalin (10%), embedded in paraffin, sectioned at an appropriate thickness (5 μm), and stained with hematoxylin and eosin (H&E) according to standard techniques. Finally, the stained slides were viewed under a light microscope and photographed using a LEICA digital camera (DFC290 HD, Switzerland).

### Data analysis and statistics

All experiments were conducted in triplicate, and the findings are shown as mean ± standard deviation. One-way ANOVA with Tukey post hoc test, as incorporated into the aov function in the R program, was used to examine statistical differences (version 3.4.4, 2018). Statistical significance was defined as *p*-values less than .05.

## Results and discussion

### Experimental design and optimization

**Figure 1.**
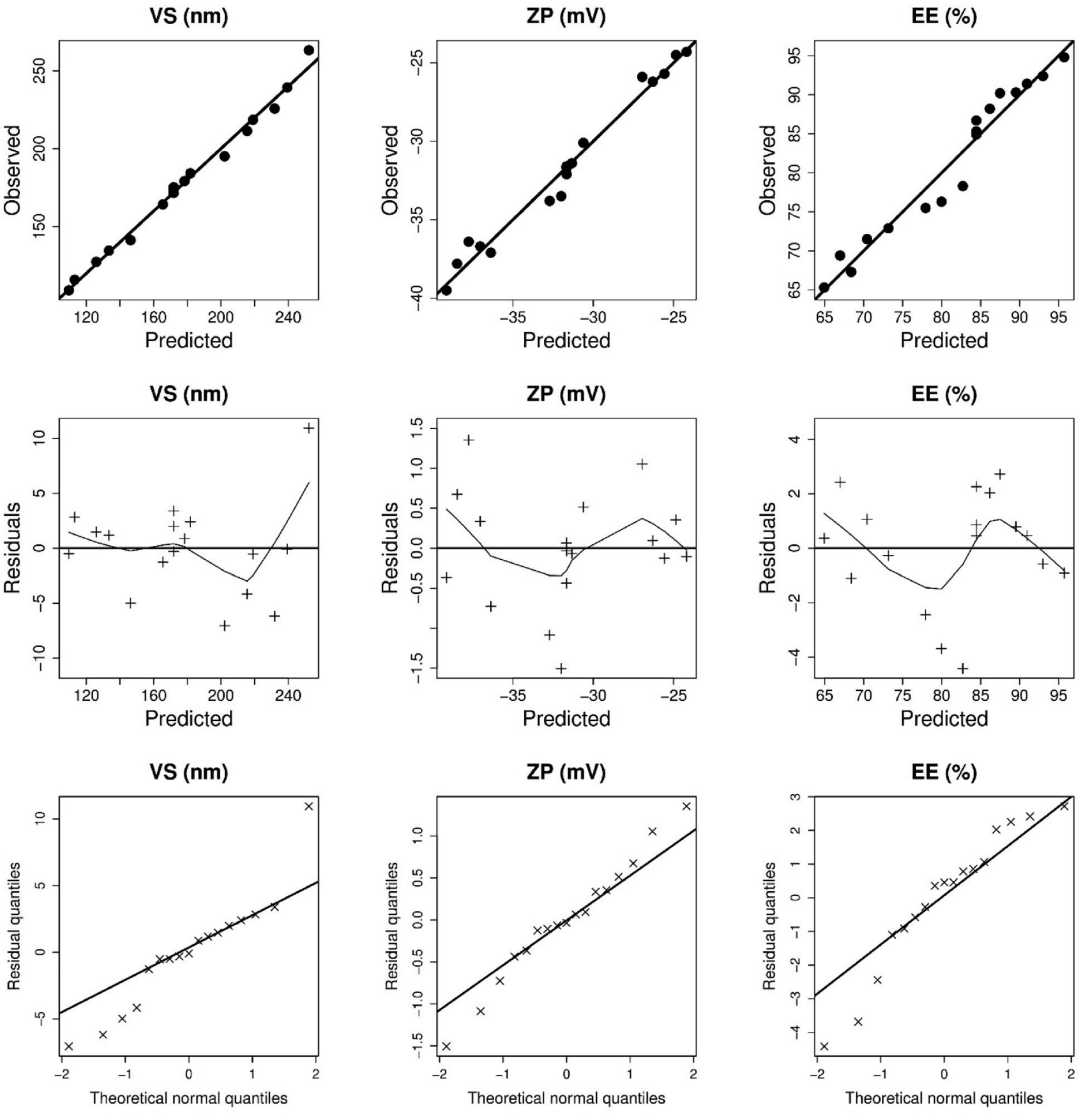
The diagnostic graphs of the dependent variables (vesicles size (VS), zeta potential (ZP), and entrapment efficiency (EE)) models.

**Figure 2.**
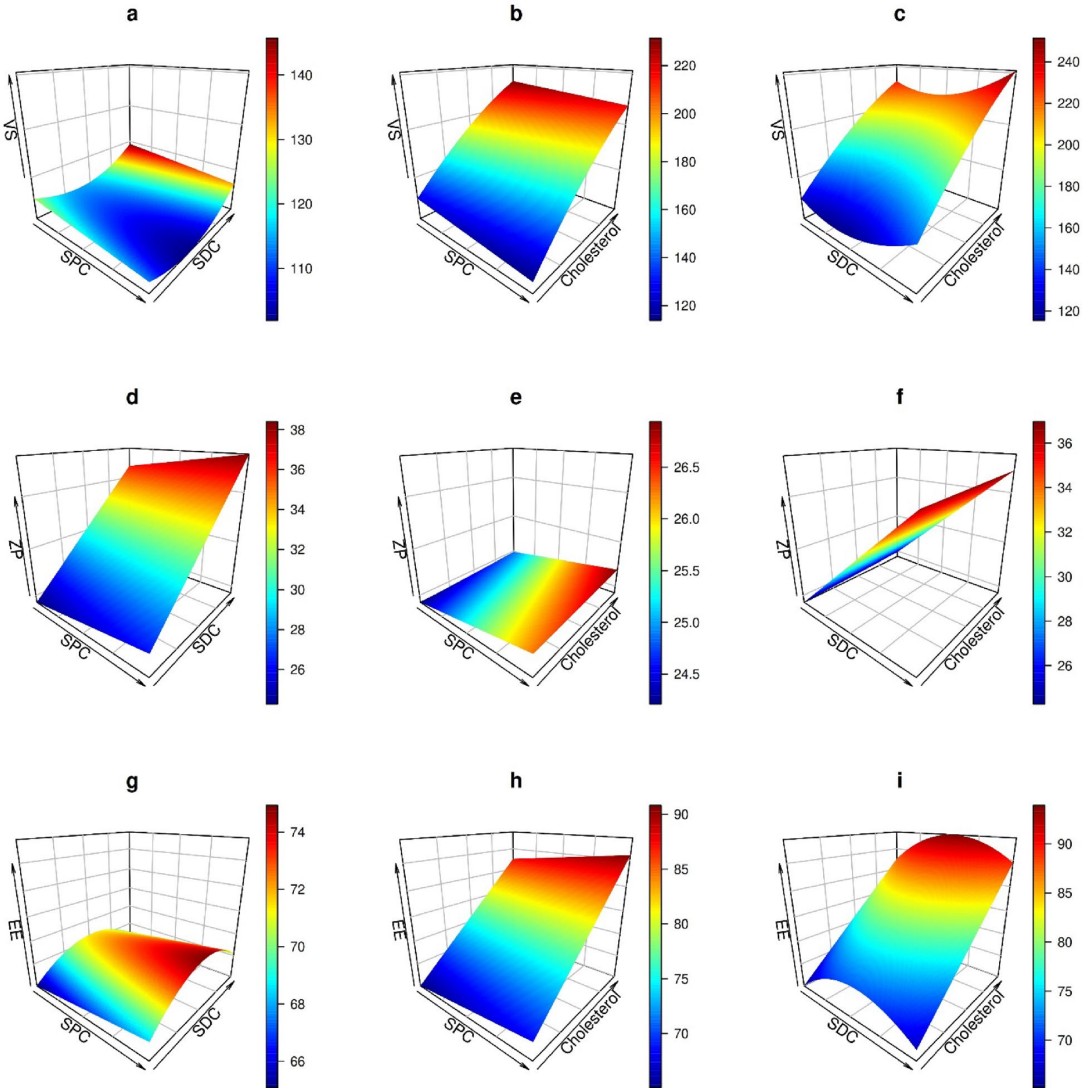
Three-dimensional response surface plots showing the effect of the independent variables (SPC, SDC, and cholesterol) on vesicle size (a, b, and c), zeta potential (d, e, and f), and entrapment efficiency (g, h, and i).

A central composite design is employed to determine and analyze the factors affecting the characteristics of a delivery system. The variables specified and their levels were selected according to exploratory trials to detect the possible independent variables. Upon analysis, the predicted *R*^2^ values were close to the adjusted *R*^2^ (Table 3), indicating that the model is adequate. Furthermore, as illustrated in Figure 1, the model diagnostic plots revealed a good fit with no noticeable residual error clusters and normal distribution. The effect of SPC molar concentration, SDC amount, and cholesterol on the VS, EE, and ZP of vesicles is graphically shown in three-dimensional response surface plots (Figure 2), and the results are presented in Tables 2 and 3.

**Table 3.** Output data of central composite design analysis.

Source	VS (nm)		ZP (mV)		EE%	
	*F*	*p*-value	*F*	*p*-value	*F*	*p*-value
Model	365.29	<0.0001	201.32	<0.0001	70.86	<0.0001
*X*_1_: SPC molar concentration	23.43	0.0005	17.39	0.0011	5.71	0.0342
*X*_2_:SDC amount (mg)	57.45	<0.0001	584.86	<0.0001	2.89	0.1149
*X*_3_: Cholesterol amount (mg)	1669.75	<0.0001	1.72	0.2127	246.41	<0.0001
*X*_2_²	73.31	<0.0001			28.44	0.0002
*X*_3_²	33.25	0.0001				
Lack of fit	5.63	0.1600	10.52	0.0899	8.46	0.1103
Model	Reduced Quadratic		Linear		Reduced Quadratic	
Adjusted *R* ^2^	0.9913		0.9741		0.9458	
*R* ^2^	0.9940		0.9789		0.9594	
%CV	1.17		2.51		1.43	
Predicted *R* ^2^	0.9836		0.9639		0.9189	
Adequate precision	58.3942		38.6770		25.5364	
SD	0.1552		0.7963		0.0016	

$\mathrm{Sqrt}(\mathrm{VS})=13.11-0.237.X_{1}+0.372.X_{2}+2.01.X_{3}+0.763.X_{2}^{2}-0.514.$.$X_{3}^{2}$

$\mathrm{ZP}=-31.66-1.05.X_{1}-6.09.X_{2}-0.33.$.$X_{3}$

$1/\mathrm{Sqrt}(\mathrm{EE})=0.11-0.001.X_{1}-0.0009.X_{2}-0.008.X_{3}+0.0042$.${.X}_{2}^{2}$

### Effect of independent variables on VS

The small size of nanovesicles is critical for intestinal penetration. As depicted in Table 2, VS ranged from (109.1 ± 2.1 to 263.2 ± 3.6 nm). ANOVA analysis showed that a reduced quadratic model was suitable, and SPC molar concentration, SDC amount, and cholesterol amount significantly affected VS (*p* < .05). The coded equation of VS is illustrated in Table 3.

Unexpectedly, increasing SPC levels resulted in a minor reduction in the VS of the BER-BLS, as seen in Figure 2(a, b). The increased surface area may explain these observations at high lipid concentrations, allowing the incorporation of more BER into the bilayer vesicle (Ahmed, 2015; Avadhani et al., 2017). The SDC level negatively influenced VS, as seen in Figure 2(a, c). The VS decreases as the SDC level rises due to decreased surface tension. Reports have indicated that SDC tends to self-aggregate at high levels, resulting in an increase in VS (Yang et al., 2019).

As shown in Figure 2(b, c), when cholesterol levels rise, VS rises significantly. The observed increase in VS might be ascribed to the increased dispersion of cholesterol molecules inside the phospholipid bilayer (Shaker et al., 2017). Furthermore, the elevated cholesterol impeded in the lipid packing of the vesicle leads to greater dispersion of the aqueous phase inside the vesicle and hence an increase in the VS (Al-Mahallawi et al., 2015).

### Effect of independent variables on ZP

The value of ZP is used to speculate the stability of colloidal dispersions in terms of electric repulsion. Typically, the formulation with a surface charge of more than 30 mV (absolute value) is expected to be physically stable (Eid et al., 2021). The values of ZP ranged from −39.5 ± 3.6 to −24.3 ± 2.8, indicating that the formulations had enough surface charge to hinder particle aggregation. ANOVA analysis revealed that a linear model was appropriate and that the levels of SPC, SDC, and cholesterol significantly influenced ZP (*p* < .05). Table 3 illustrates the ZP equation in its coded form.

Figure 2(d, e) showed that the negative charge on the vesicles rises as SPC levels increase. This rise in the negative charge has resulted from the negative phosphate groups (PO3^−2^) that exist in SPC (Souza et al., 2014). Additionally, since SDC is negatively charged, a high SDC content increases the ZP negative charge of the vesicles, as seen in Figure 2(d, f) (Abdellatif et al., 2017).

### Effect of independent variables on EE

Entrapment of high amounts of medications is a notable aspect of BLS that contributes to its potential application as an oral delivery system. The entrapment efficiency ranged between 65.3 ± 2.5 and 94.8 ± 4.7%, as reported in Table 2. ANOVA analysis showed that a reduced quadratic model was suitable, and SPC molar concentration and cholesterol amount significantly affected entrapment (*p* < .05). The coded equation of EE is illustrated in Table 3.

Increases in SPC molar concentration enhance the lipophilicity of the vesicular system, hence raising the entrapment, as demonstrated in Figure 2(g, h) (Abdelbary & Aburahma, 2015). While SDC level had little influence on entrapment, as seen in Figure 2(g, i), some previous reports indicated that BLS entrapment has decreased as the SDC concentration has increased (Aburahma, 2016).

Notably, cholesterol was shown to have a synergistic impact on entrapment efficiency, i.e., increasing cholesterol results in greater entrapment, as seen in Figure 2(h, i). Cholesterol has been shown to augment the hydrophobicity and stiffness of lipid bilayer membranes. As a result, it improves the stability of the BLS and inhibits medication leakage (Jain et al., 2014; Qumbar et al., 2017).

### Selection of optimized BER-BLS formulation

The mathematical optimization approach of Design Expert® software was used to select the optimum formulation based on the criteria of maximizing the values of EE and ZP and minimizing the value of the VS. The formulation with SPC molar concentration of 0.06, SDC amount of 25 mg, and cholesterol amount of 15.2 mg was determined to meet the requirements of an optimum formulation after ‘trading off’ various responses using a desirability function. Point prediction demonstrated a low prediction error (<12%) for each of the measured responses (Table 4).

**Table 4.** Experimental, predicted, and prediction error values of dependent variables of the optimized BER-BLS formulation.

	VS (nm)	ZP (mV)	EE%
Experimental value	196.5	(−) 36.4	89.7
Predicted value	173.3	(−) 34.8	86.2
Prediction error (%)^a^	11.8	4.4	3.9

a Computed as (Experimental value − Predicted value)/Experimental value ×100.

**Figure 3.**
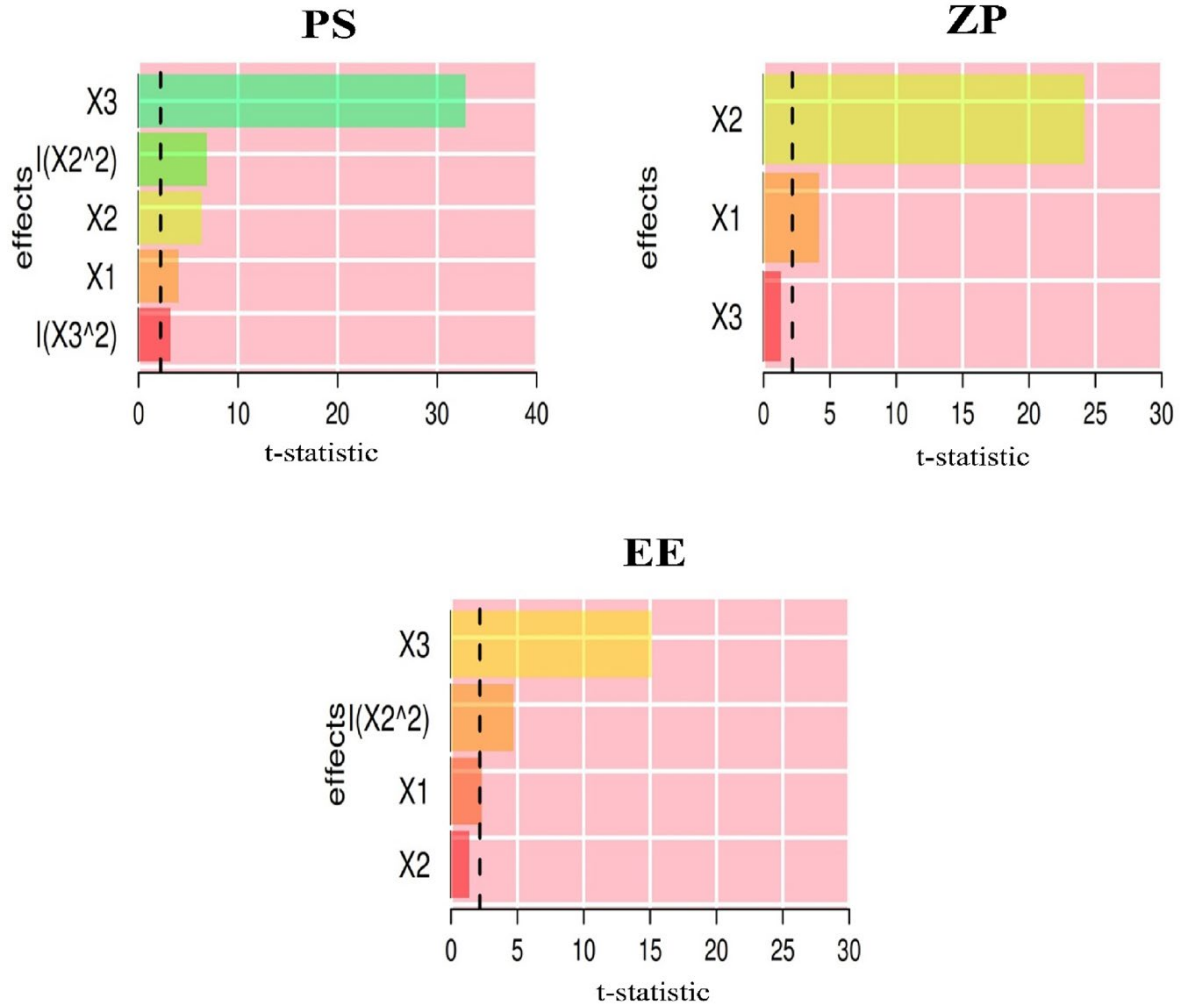
Pareto chart depicting the standardized impacts of independent variables; SPC molar concentration (*X*_1_), SDC amount (*X*_2_), and Cholesterol amount (*X*_3_).

Pareto chart (Figure 3) showed the standardized relative impacts of the formulation factors on VS, ZP, and EE%. VS and EE% were more influenced by cholesterol than by SPC and SDC levels. However, the SDC level was the most influential for ZP.

### Optimized BER-BLS characterization

#### Transmission electron microscopy

**Figure 4.**
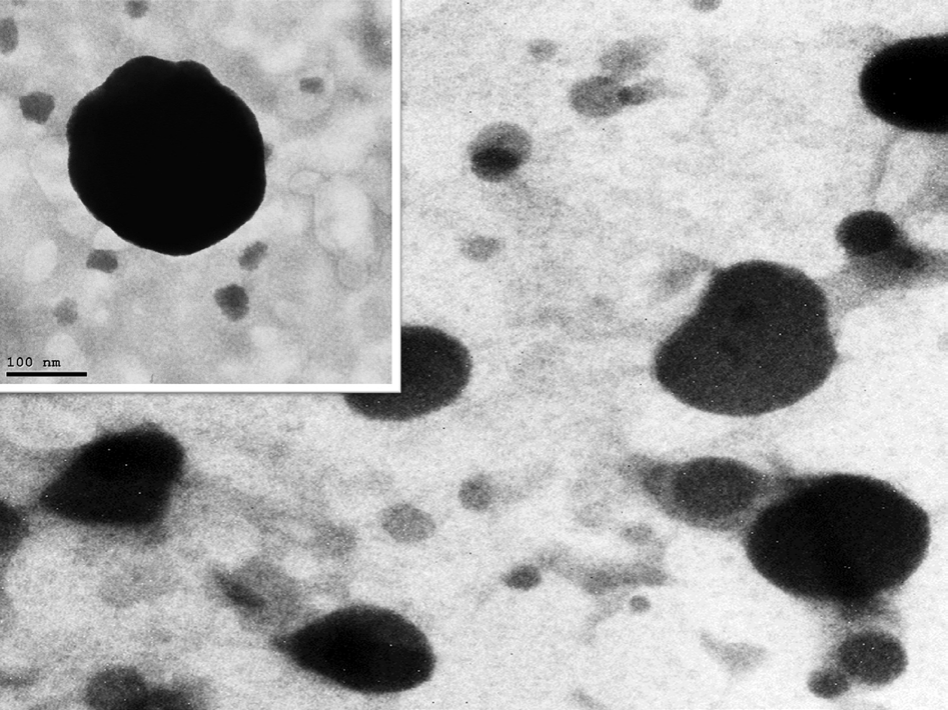
The TEM micrograph of the optimized BER-BLS formulation.

Figure 4 illustrates the morphology of the optimized BER-BLS formulation. The TEM image of the optimized BER-BLS showed spherical unilamellar vesicles devoid of vesicular aggregation. In addition, the average VS of the optimized formulation was likewise consistent with the dynamic light scattering (DLS) findings.

#### In vitro release study

**Figure 5.**
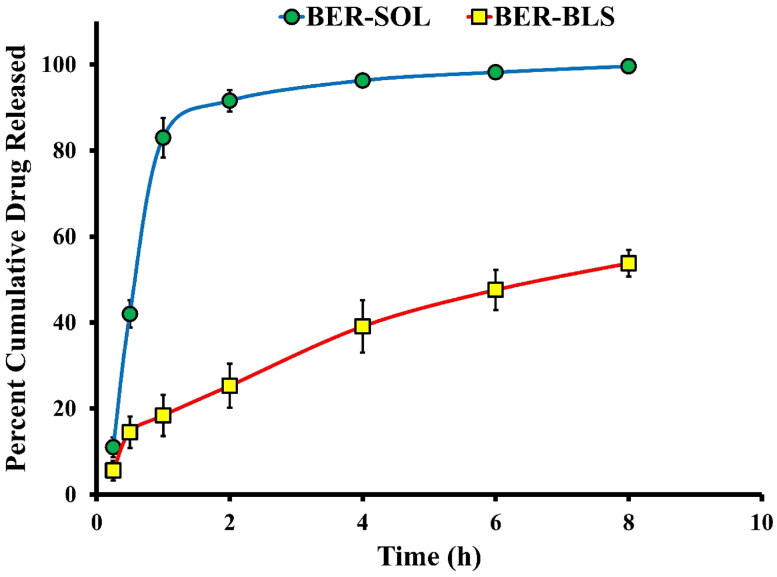
*In vitro* BER release profile from the optimized BER-BLS and BER-SOL.

*In vitro* release has been considered a critical surrogate indication of in vivo efficacy, particularly for poorly water-soluble drugs. Figure 5 compares the *in vitro* release profiles of BER-SOL and optimized BER-BLS. The release of BER-SOL was rapid, with roughly 91.6% released within 2 h. This observation may be explained by the fact that BER in a solution can diffuse rapidly over the dialysis membrane, demonstrating that the dialysis membrane has a minimal hindering effect on BER release. In comparison, the release of BER from the optimized BER-BLS formulation seemed to be much slower, with a maximum of 40% at 4 h and less than 54% at 8 h. The high affinity of BER for the hydrophobic components in the bilosomal formulation was therefore attributed as a probable explanation for the slow BER release from the optimized bilosomal formulation (Guan et al., 2011). The surface-active property of SDC seems ineffective, although treating with surfactants has been reported as a powerful approach to hydrophilizing systems and increases their proclivity for dissolving in aqueous media (Elkomy et al., 2021).

#### Stability study

**Figure 6.**
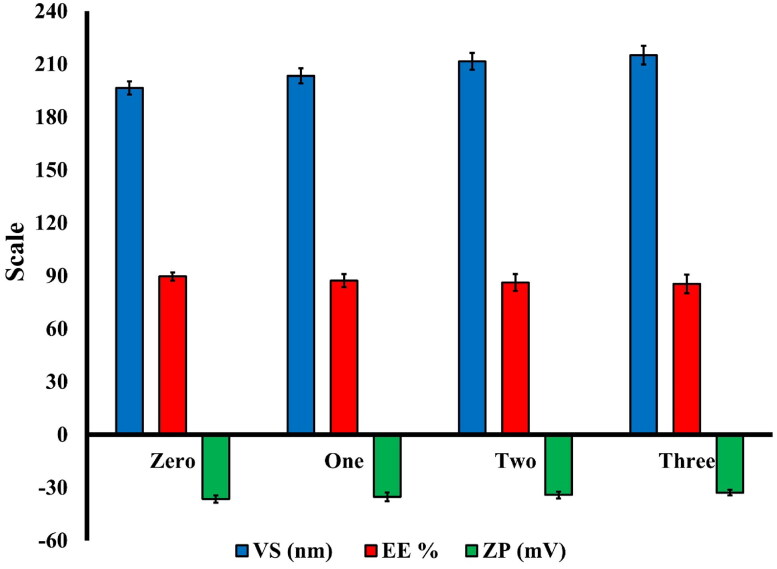
The VS, EE, and ZP of the optimized BER-BLS formulation after three months of storage.

Following 90 days of storage, the optimized BER-BLS formulation showed a homogenous appearance with no aggregation. The changes in the VS, EE%, and ZP of the stored optimized BER-BLS formulation are illustrated in Figure 6. There were no significant changes in VS, %EE, and ZP of the kept samples during the storage period (*p* > .05). The high stability of the optimized BER-BLS may be attributed to the high negative surface charge that resulted from the anionic nature of SDC (Abdelbary et al., 2016) and the nanoscale of the optimized bilosomal formulation.

### In vivo experiments

#### Hypoglycemic effect in diabetic rats

**Figure 7.**
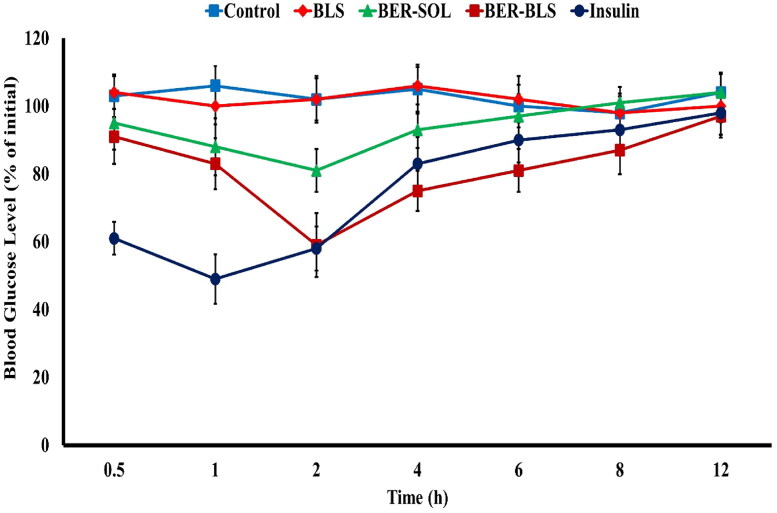
Blood glucose levels (% of initial) after oral administration of saline, BLS (blank carrier), BER-SOL, and BER-BLS and subcutaneous insulin administration as a positive control. Data represented as mean ± SD.

Figure 7 depicts the blood glucose levels versus time after administration of several formulations to streptozocin-induced diabetic rats. After drug administration, significant differences in plasma glucose decline (% relative to the starting value) were reported between the control, BER-SOL, BER-BLS, and insulin groups (*p <* .05). BER-SOL had a negligible hypoglycemic impact, with a maximum decline in blood glucose of just 19% compared to the saline group. In contrast, BER-BLS had a more substantial hypoglycemic impact, with a maximum blood glucose reduction of 41%. As a result, BER-BLS resulted in approximately a double decrease in blood glucose relative to the solution formulation, showing that more BER molecules were transported into the systemic circulation through BLS. Furthermore, when compared to insulin (1 IU), the pharmacological effect (PE) of oral BER-BLS and BER-SOL was 99.3% and 31.7%, respectively, according to the AAC. Understandably, the effectiveness of BER-BLS in decreasing blood glucose was less than that of subcutaneous insulin. Undoubtedly, BER-BLS are more successful than BER-SOL in regulating blood glucose, as they avoid rapid fluctuations in blood glucose and provide a long-lasting pharmacological impact.

#### Bioavailability and pharmacokinetic study

**Figure 8.**
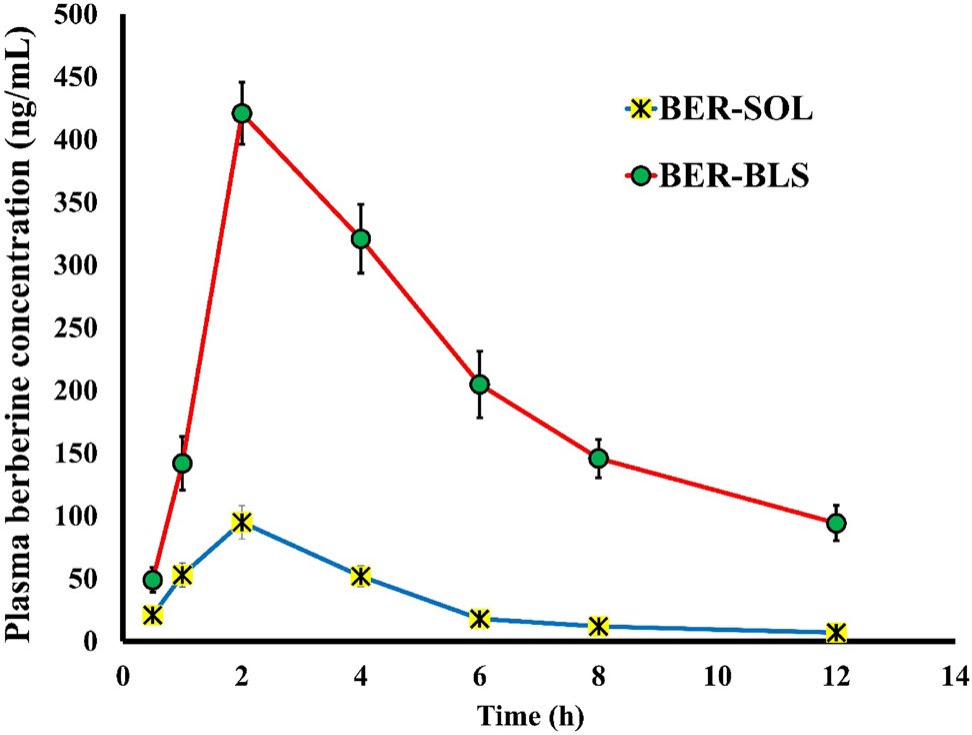
Plasma concentration of berberine following oral administration of BER-SOL and BER-BLS in diabetic rats. Data represented as mean ± SD.

Figure 8 depicts the pharmacokinetic profiles of BER-SOL and BER-BLS after oral administration. BER-SOL was absorbed poorly and had low oral bioavailability. In comparison to BER-SOL, BER-BLS dramatically increased drug absorption in both rate and extent. Table 5 summarizes the major pharmacokinetic characteristics of BER-SOL and BER-BLS. The maximum plasma concentration (*C*_max_) and area under the curve (AUC_0–t_) for BER-SOL were 95 ± 13.26 ng/mL and 382.75 ± 47.2 ng⋅h/mL, respectively. In contrast, BER-BLS provided a substantially higher BER plasma level and maintained it for a long time beyond the peak period. The *C*_max_ and AUC_0–t_ levels were 421 ± 24.73 ng/mL and 2441.1 ± 95.3 ng⋅h/mL, respectively. The findings indicate that the bilosomal delivery strategy remarkably enhanced BER oral absorption. The increased *C*_max_ of BER-BLS is ascribed to its nanoscale diameter, high entrapment and stability in GIT fluid, and superior permeability. In addition to the fact that it bypasses first-pass metabolism. Besides, the high AUC value is due to the gradual and extended release of BER, which assists in the absorption of the maximal quantity of BER. Comparing BER-BLS to BER-SOL, the relative oral bioavailability of BER-BLS was 637.7% using the non-compartmental model. While BER-SOL and BER-BLS had an equal *in vivo* half-life (*t*_1/2_ = 4.5 h), they markedly had different mean residence times (MRT), as seen in Table 5. Overall, the data of pharmacokinetic analysis demonstrate that the BLS significantly increases the relative bioavailability of BER-BLS ∼ 6.4-fold compared to BER-SOL. The improved bioavailability might be attributed to greater BLS absorption by Peyer’s patch intestinal M-cells and increased solubility in the presence of lipid and surfactant (Elkomy et al., 2021). Additionally, the ultra-deformability of BLS may facilitate absorption through carrier-mediated transmembrane transport (Elkomy et al., 2022). These features make BLS an excellent vehicle for the oral administration of berberine.

**Table 5.** Pharmacokinetic parameters of BER in Wistar rats after oral delivery of BER-SOL and BER-BLS.

Parameter	BER-SOL	BER-BLS
*C*_max_ (ng/ml)	95 ± 13.26	421 ± 24.73
*T*_max_ (h)	2 ± 0.45	2 ± 0.61
*T*_1/2_ (h)	4.5 ± 0.71	4.5 ± 0.84
AUC_0-t_ (ng. h/ml)	382.75 ± 47.2	2441.1 ± 95.3
Mean residence time (h)	5.27 ± 0.87	7.69 ± 0.59
Relative bioavailability (%)	–	637.8

#### In vivo histopathological study

**Figure 9.**
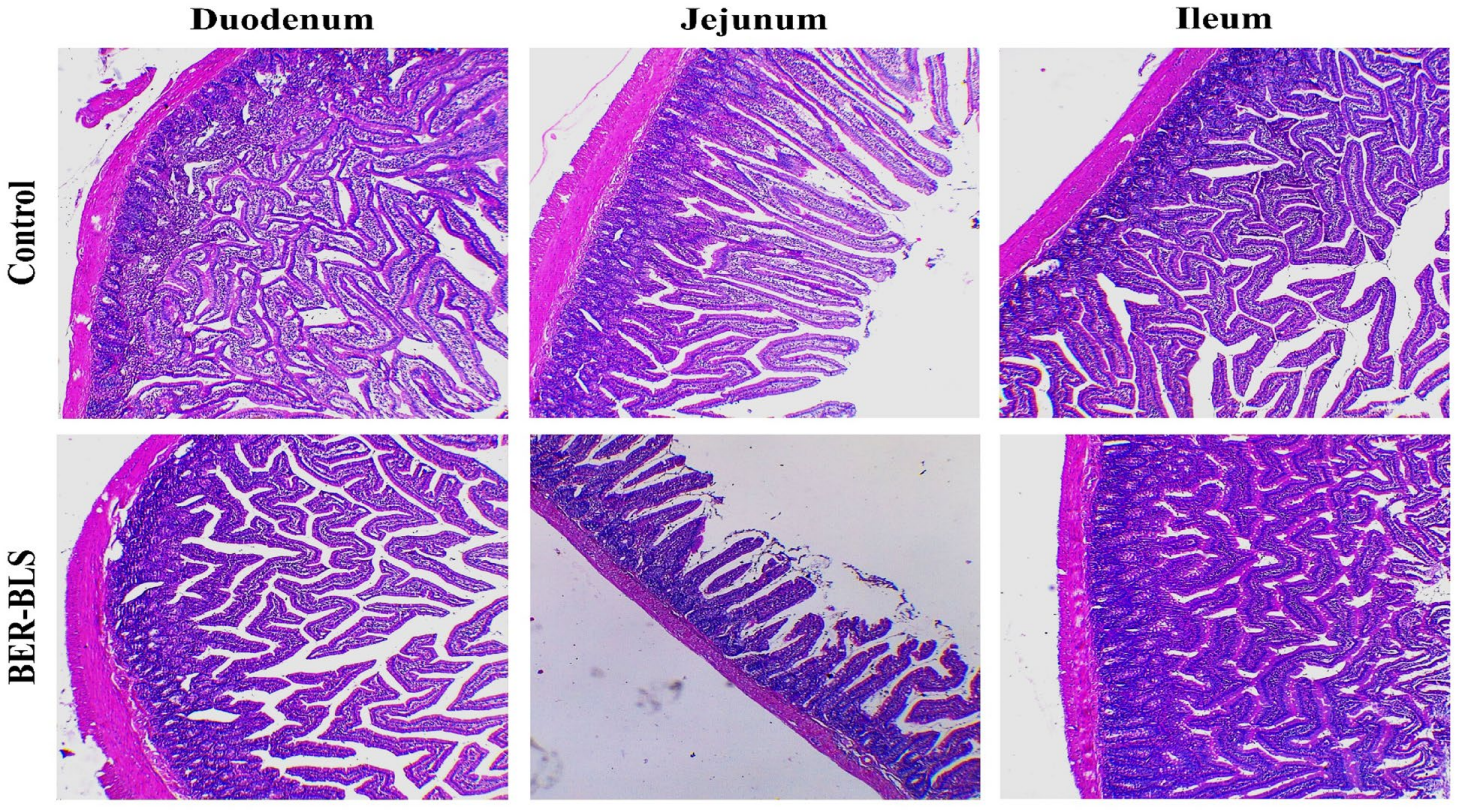
Hematoxylin and eosin-stained small intestinal segments of Wistar rats (Duodenum, Jejunum, and Ileum) after 24 h from oral administration of normal saline solution (A) and the optimized BER-BLS formulation.

Histological analyses of tissue were employed to examine the pathological alterations produced by BLS in the small intestinal epithelium. The microscopic appearance of the intestinal epithelium sections (duodenum, jejunum, and ileum) following 24 h from oral administration of the optimized BER-BLS formulation was comparable to the control group. The results revealed no inflammatory cells, necrosis, or signs of villi damage (Figure 9).

## Conclusions

We successfully developed a bilosomal system for the oral administration of berberine to increase its bioavailability and hypoglycemic effects. The proposed BER-BLS had a prolonged releasing effect with high stability. The bilosomal oral delivery significantly enhanced the pharmacokinetics of berberine and boosted its hypoglycemic impact. This innovative BLS-based BER delivery system may be an oral alternative to perorally and parenterally administered antidiabetic agents.
